# Rice-SVBDete: a detection algorithm for small vascular bundles in rice stem’s cross-sections

**DOI:** 10.3389/fpls.2025.1589161

**Published:** 2025-05-26

**Authors:** Xiaoying Zhu, Weiyu Zhou, Jianguo Li, Mingchong Yang, Haiyu Zhou, Jiada Huang, Jiahua Shi, Jun Shen, Guangyao Pang, Lingqiang Wang

**Affiliations:** ^1^ Guangxi Colleges and Universities Key Laboratory of Intelligent Software, Wuzhou University, Wuzhou, China; ^2^ State Key Laboratory of Conservation and Utilization of Subtropical Agricultural Biological Resources, College of Agriculture, Guangxi University, Nanning, China; ^3^ Guangxi Key Laboratory of Sugarcane Biology, College of Agriculture, Guangxi University, Nanning, China; ^4^ Centre for Nutrition and Food Sciences, Queensland Alliance for Agriculture and Food Innovation, The University of Queensland, Brisbane, QLD, Australia; ^5^ School of Computing and Information Technology, University of Wollongong, Wollongong, NSW, Australia; ^6^ Centre for Crop Science, Queensland Alliance for Agriculture and Food Innovation, The University of Queensland, Brisbane, QLD, Australia

**Keywords:** rice vascular bundles, small object detection, deformable convolution, deep learning, YOLO

## Abstract

**Introduction:**

Vascular bundles play a vital role in the growth, development, and yield formation of rice. Accurate measurement of their structure and distribution is essential for improving rice breeding and cultivation strategies. However, the detection of small vascular bundles from cross-sectional images is challenging due to their tiny size and the noisy background typically present in microscopy images.

**Methods:**

To address these challenges, we propose Rice-SVBDete, a specialized deep learning-based detection algorithm for small vascular bundles in rice stem cross-sections. Our approach enhances the YOLOv8 architecture by incorporating three key innovations: Dynamic Snake-shaped Convolution (DSConv) in the Backbone network to adaptively capture intricate structural details of small targets. A Multi-scale Feature Fusion (MFF) mechanism, combining features from the Backbone, Feature Pyramid Network (FPN), and Path Aggregation Network (PAN), to better handle objects at multiple scales. A new Powerful Intersection over Union (PIoU) loss function that emphasizes spatial consistency and positional accuracy, replacing the standard CIoU loss.

**Results:**

Experimental evaluations show that Rice-SVBDete achieves a precision of 0.789, recall of 0.771, and mean Average Precision (mAP@.5) of 0.728 at an IoU threshold of 0.50. Compared to the baseline YOLOv8, Rice-SVBDete improves precision by 0.179, recall by 0.201, and mAP@.5 by 0.227, demonstrating its effectiveness in small object detection.

**Discussion:**

These results highlight Rice-SVBDete's potential for accurately identifying small vascular bundles in complex backgrounds, providing a valuable tool for rice anatomical analysis and supporting advancements in precision agriculture and plant science research.

## 1 Introduction

Vascular bundles play a vital role in water conduction, the transport of inorganic salts and organic nutrients, and mechanical support within plants. In rice stems, parameters such as the number, area, ratio, and distribution of large and small vascular bundles are critical intrinsic structural factors that influence the physicochemical properties and functional characteristics of the stem Li et al. (2024a). Anatomical analysis of stem features provides deeper insights into key biological traits, including the processes of stem growth and development, environmental adaptability, and stress resistance Bapat et al. (2023). Deciphering the genetic basis of structural traits in crop stems and identifying related gene resources are of great significance for the genetic improvement of crop lodging resistance and yield traits.

Object detection is a critical application in the traditional field of computer vision, where methods based on Convolutional Neural Networks (CNNs) have achieved remarkable progress and breakthroughs in recent years Wu et al. (2020); Zou et al. (2023). With the rapid development and widespread adoption of information technology, leveraging advanced artificial intelligence techniques for efficient and accurate automated detection of rice stem cross-sectional parameters plays a crucial role in tasks such as crop breeding, precision agricultural management, and pest and disease diagnosis. However, detecting small vascular bundles presents unique and challenging characteristics, such as highly variable morphology, dense arrangement, indistinct edges, and low contrast. These, combined with the inherent challenges of small object detection, such as small size, low resolution, and vulnerability to noise, make the detection of small vascular bundles in rice stem’s cross-sections a particularly demanding task.

The rapid development of deep learning technologies has offered new solutions for small object detection. Currently, mainstream object detection algorithms are primarily divided into region proposal-based two stage detection algorithms and regression-based one-stage detection algorithms. Two-stage detection algorithms Girshick et al. (2014); Ren et al. (2016) extract candidate regions and features through CNNs before performing classification and boundary regression for fine-grained object detection. While these methods achieve high detection accuracy, they also result in lower efficiency and a higher false positive rate. In contrast, one-stage detection algorithms Liu et al. (2016); Ross and Dollár (2017); Hussain (2023) bypass the generation of proposal boxes and directly extract features within the network to predict object classification and location. Compared to two-stage detection algorithms, one-stage methods are faster. Among them, the You Only Look Once (YOLO) series algorithms Hussain (2023); Wang et al. (2024); Khanam and Hussain (2024) strike a better balance between detection accuracy and computational cost. However, despite the significant achievements of the YOLO series across various fields, there remains room for improvement in small object detection. The primary challenges stem from insufficient feature representation for small objects and the inadequacies of existing functional loss designs for small object detection.

To address the challenges of small object detection, researchers have explored methods to capture more feature information within the network, improve network structures, and minimize feature loss during transmission, thereby enhancing the detection capabilities for small objects. Zhu et al. (2020) proposed a deformable end-to-end object detection framework called DERT, which incorporated a built-in deformable attention module and is equipped with a deformation-enhanced FPN network that requires no additional support. By leveraging attention mechanisms to fuse multi-scale features, DERT achieves significant improvements in convergence speed while maintaining high performance, excelling particularly in small object detection tasks. Chen et al. (2024a) proposed a small object detection model for drone aerial images (SOD-YOLOv7) based on the real-time detector YOLOv7. While this improved method effectively maintained focus on small objects, the real-time detector significantly increased the parameter count of the model, reducing computational speed. Wu et al. (2022) introduced an Intersection over Union (IoU) balanced loss function for single-stage object detection, aiming to balance classification loss. This encourages the model to focus more on high-IoU positive samples, enhancing the correlation between classification and localization tasks to improve localization accuracy. Zhao et al. (2024) developed a detection algorithm for tiny and complex objects in drone aerial images (Subtle-YOLOv8). Subtle-YOLOv8 incorporates Dynamic Snake Convolution (DSConv) and a Multi-scale Attention Module (EMA) into the original YOLOv8 network to enhance its detection capability for tiny objects. However, introducing DSConv and EMA increases the model’s computational complexity and memory usage. Chen and Zhang (2024) proposed an innovative cross-scale feature fusion method (HEPAN), which adds a SCDown down-sampling module to the network. This approach significantly reduces model parameters and computational complexity without compromising detection capability. Zhu et al. (2024) presented a single-point supervised detection method for tiny objects, which decomposes learning into two stages to address label noise caused by scale ambiguity and positional offset in point annotations. However, this process relies heavily on the quality of the coarse pseudo-boxes generated in the first stage. If the pseudo-boxes are inaccurate, the second stage might fail to refine them effectively.

In addition to YOLO-based approaches, a growing body of work has investigated more flexible and adaptive architectures for small object detection, especially in the infrared domain. Zhang et al. (2024) proposed IRSAM, an enhanced Segment Anything Model that incorporates a Perona-Malik diffusion block and a granularity-aware decoder to bridge the domain gap between natural and infrared imagery, thereby improving the representation of small targets. Chen et al. (2024b) introduced MiM-ISTD, a hierarchical structure that treats local image patches as “visual sentences” and decomposes them into “visual words” using a Mamba-in-Mamba architecture. This formulation allows for efficient local feature representation and delivers state-of-the-art performance on multiple infrared datasets. Transformer based methods have also seen rapid progress. Yuan et al. (2024) presented SCTransNet, a spatial-channel cross transformer that enhances feature discrimination between small targets and cluttered backgrounds. Similarly, Li et al. (2024b) designed a lightweight Transformer-based decoder with high-frequency aware modules to integrate global context with fine-grained details. Furthermore, adaptive and contrast-based models have emerged. Li et al. (2024c) proposed an iterative threshold analysis combined with adaptive region growing for better target localization. Chen et al. (2024c) incorporated deformable attention mechanisms with cross-aggregation strategies guided by local contrast priors. These methods highlight the increasing use of attention and hybrid CNN-Transformer modules to address challenges posed by small target size, low contrast, and complex backgrounds. These recent developments suggest that integrating multi-scale context modeling, deformable attention, and adaptive region priors will be critical to improving small object detection tasks in the future.

Given the YOLO series algorithms’ lightweight nature, efficiency, and rapid processing capabilities, we herein propose a new algorithm, Rice-SVBDete, based on YOLOv8. The primary contributions of our work in this field are as follows:

- We propose a new strategy to enhance feature extraction capabilities by incorporating Dynamic Snake Convolution (DSConv) Zhang et al. (2020) into the Backbone network. This integration improves the model’s precision in identifying the subtle boundary structures of small vascular bundles, enabling the model to better capture fine and intricate details.
- We designed a Multi-scale Feature Fusion method (MFF) by incorporating additional Upsample, C2f, and Concat modules into the feature pyramid network of the Neck network and adding a new detection head to the original network structure. This approach significantly enhances the model’s capability to represent multi-scale features, enabling a more precise capture of fine-grained object characteristics. While improving segmentation accuracy, the method also effectively boosts the model’s robustness and adaptability to segmentation tasks across different scales.
- We replaced the original Complete Intersection over Union (CIoU) loss function with the Powerful Intersection over Union (PIoU) loss function to better optimize the model’s performance in small object detection. PIoU effectively enhances the spatial matching between predicted and ground truth boxes by introducing a dedicated penalty term, demonstrating significant advantages in handling small, dispersed objects. Additionally, the design of PIoU simplifies the computational process, requiring only a single hyperparameter to adjust the weight distribution of the loss function. This facilitates the acceleration of model convergence and optimization performance while simultaneously achieving an improved balance between detection accuracy and segmentation quality, thereby significantly enhancing the model’s efficiency and stability.
- We conducted a series of comparative experiments with existing state-of-the-art methods, such as YOLOv8 Jocher et al. (2023), ASF-YOLO Kang et al. (2024), SOD-YOLO Khalili and Smyth (2024), and Subtle-YOLO Zhao et al. (2024), to evaluate the performance of the Rice-SVBDete method. The experimental results show that the Rice-SVBDete method achieved higher accuracy in detecting small vascular bundles in rice stem’s cross-sections, highlighting its practical application potential in the anatomical feature analysis of rice stems.

## 2 Problem statement

**Figure 1:**
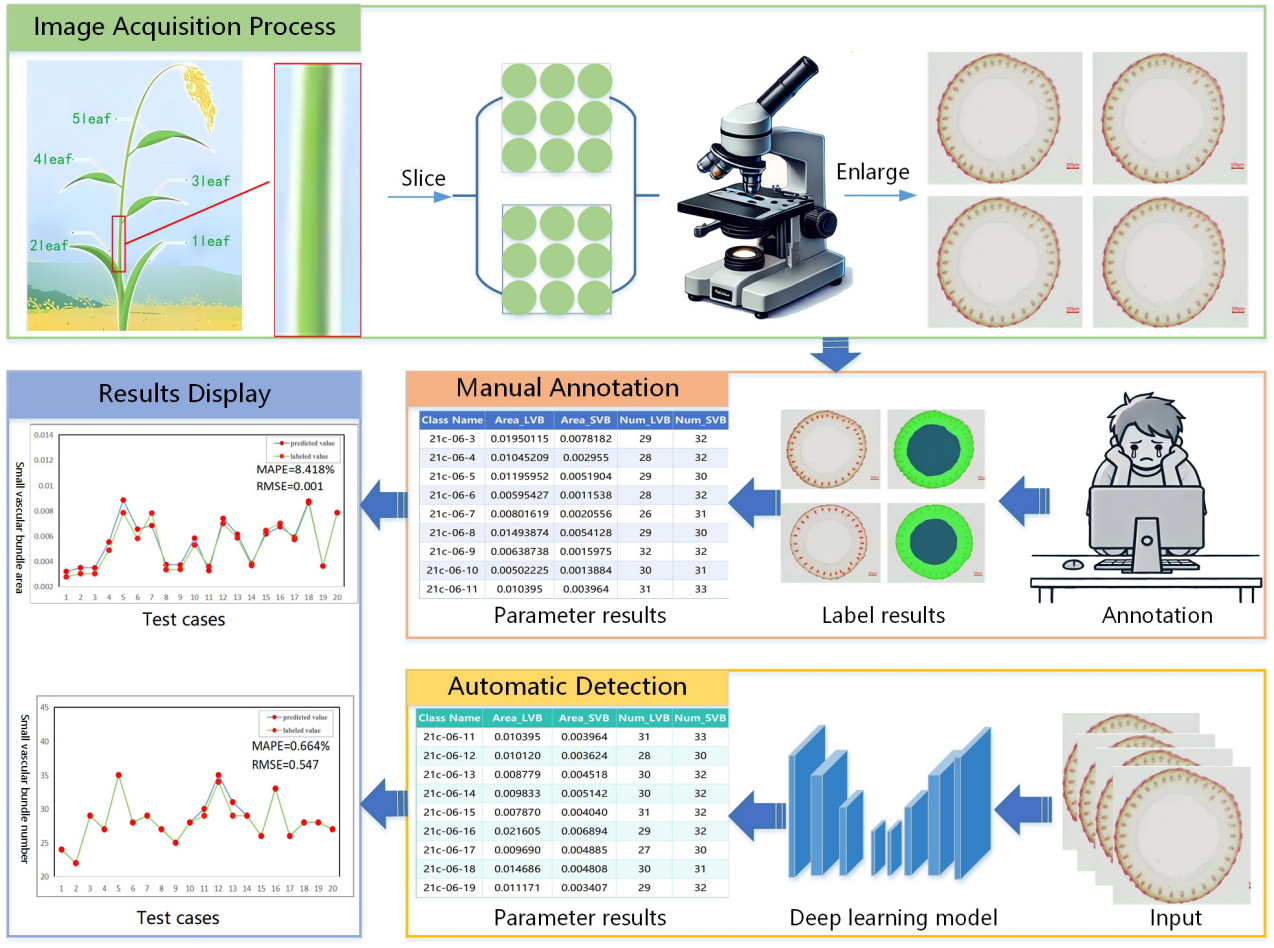
Challenges in the detection of small vascular bundles.

Traditional detection of small vascular bundles in rice stem’s cross-sections typically requires magnification and photography via using a microscope, followed by manual calculation and statistical analysis. As illustrated in **Figure 1**, each microscopic image contains numerous small vascular bundles that are both highly abundant and exceptionally tiny. This makes manual annotation prone to visual fatigue, which in turn affects both the accuracy and efficiency of the results. Since the phenotypic parameters of rice stem cross-sectional images are directly related to factors such as stem growth status, nutrient absorption capacity, and the genetic traits of the variety, we raise the following question: how can we achieve automated detection of small vascular bundles in rice stem’s cross-sections under conditions of small size, dense arrangement, blurry edges, and low contrast.

To systematically address this issue, we define the terms and symbols used in this study: given a dataset of rice stem cross-sectional microscopic images *X* and their corresponding annotations *Y*, the goal is to develop a fitted model *f* (*X*) that can accurately identify and classify small vascular bundles in new, unseen rice stem cross-sectional microscopic images.

Let $X=\{X_{1},X_{2},\ldots ,X_{i},\ldots ,X_{N}\}$ represent a dataset of rice stem cross-sectional microscopic images, where each image $X_{i}$ contains multiple unit features, and *N* denotes the total number of images in the dataset. Each target bounding box is represented as $Y=\{Y_{1},Y_{2},\ldots ,Y_{i},\ldots ,Y_{N}\}$ , where each $Y_{i}$ contains one or more bounding boxes indicating the locations of feature units in image $X_{i}$ . For each feature unit *j* in image $X_{i}$ , the bounding box is represented as $Y_{i}^{j}=\{[x_{i1}^{j},y_{i1}^{j}],[x_{i2}^{j},y_{i2}^{j}]\}$ , where $\left[x_{i1}^{j},y_{i1}^{j}\right]$ and $\left[x_{i2}^{j},y_{i2}^{j}\right]$ are the coordinates of the top-left and bottom-right corners of the bounding box, respectively.

## 3 Method

This section introduces three main modules: the Dynamic Snake Convolution module(DSConv), the Multi-scale Feature Fusion method module(MFF), and the PIoU loss function module. These modules are designed to improve vascular bundles’ detection accuracy and reliability in rice stem’s cross-sections.

### 3.1 Dynamic snake convolution module

In the detection of small vascular bundles in rice stem’s cross-sections, a series of challenges arise due to their microscopic characteristics and the complexity of biological structures. Small vascular bundles, owing to their tiny size, are susceptible to interference from lighting conditions, uneven cross-sections, background impurities, and the texture of biological tissues themselves. These fine structures necessitate that the detection algorithm should demonstrate both high sensitivity and the capability to precisely distinguish the target vascular bundles from the surrounding complex biological tissue background. For instance, accurately identifying and differentiating vascular bundle tissues with varying shapes and densities in stem cross-sections, especially under uneven lighting and variable tissue textures, presents a highly challenging task. Moreover, compared to other tissues in the stem, vascular bundles often have finer and harder-to-define boundaries.

**Figure 2:**
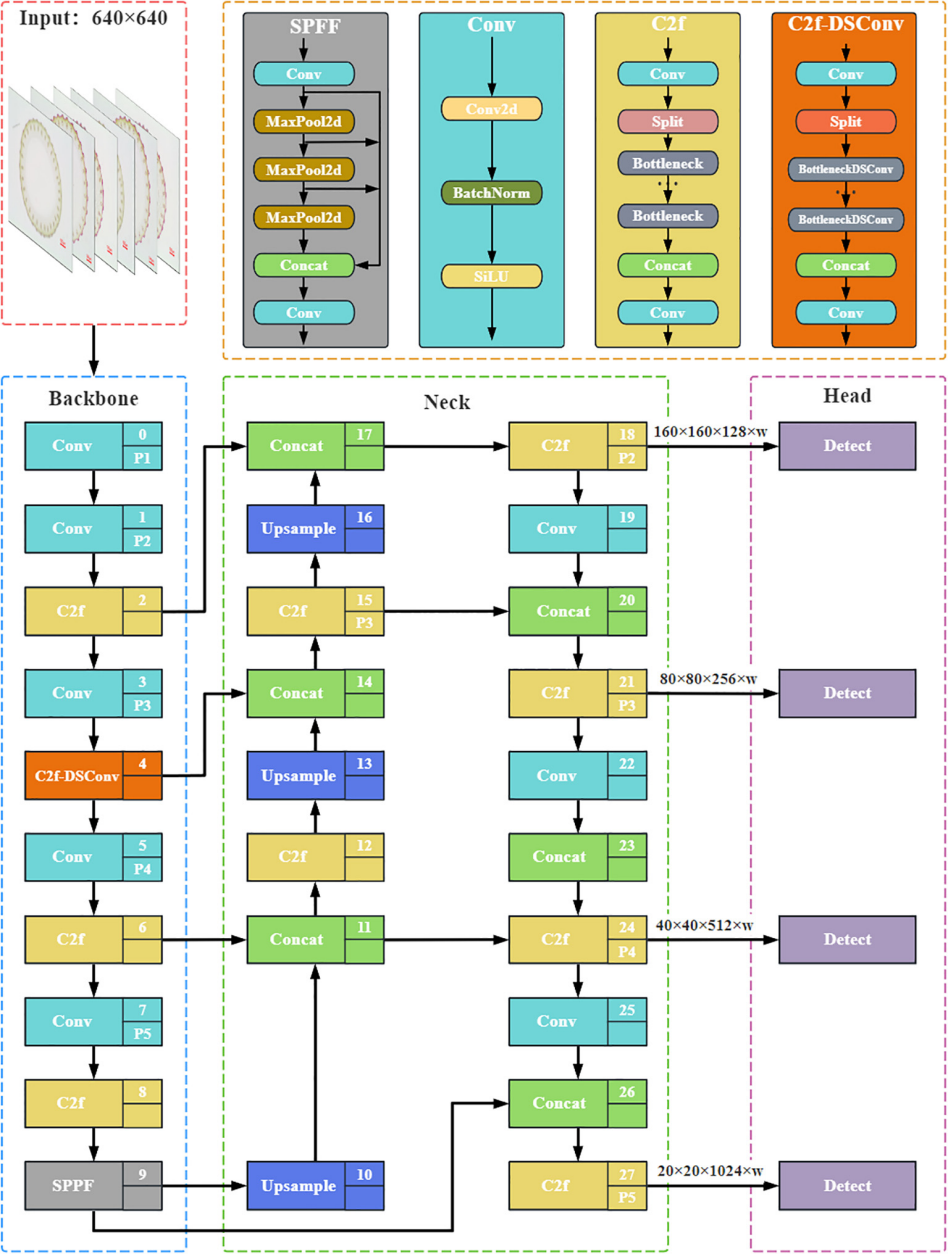
Network structure of Rice-SVBDete. C2f-DSConv module in the Backbone network improves the model’s accuracy in recognizing the fine structures at the boundary of small vascular bundles. Multi-feature fusion mechanism of Neck network accurately captures fine-grained target features.

To effectively detect small vascular bundles in rice stem’s cross-sections, we integrate DSConv into the C2f module of the Backbone network, which serves as the feature extraction network responsible for capturing and processing hierarchical features from the input images. This design aims to enhance the model’s ability to perceive small and complex structures, thereby maximizing the extraction of vascular bundle feature information from stem cross-section images. By employing this approach, we improve the model’s accuracy and robustness in detecting small vascular bundles against complex biological tissue backgrounds. Specifically, the standard convolution in the Bottleneck module, a fundamental building block designed to reduce computational cost while maintaining feature representation, is replaced with DSConv, creating an improved BottleneckDSConv module. Additionally, DSConv is used in the convolutions and is responsible for channel adjustment before and after the C2f module. As shown in **Figure 2**, the enhanced C2f module is transformed into the C2f-DSConv module, serving as a feature extraction component in the Backbone network.

DSConv was developed to address the high memory access and computational costs inherent in standard convolution operations. It achieves this by decomposing the traditional convolution operation into two parts: Vector Quantized Kernels (VQK) and Distributed Shifting. VQK quantizes the floating-point tensor weights into integers, reducing memory usage and accelerating computation speed. Distributed Shifting adjusts the values in the VQK through Kernel Distribution Shifts (KDS) and Channel Distribution Shifts (CDS), scaling and biasing the values to ensure that the output matches that of the original weight tensor.

#### 3.1.1 Quantization of VQK

VQK takes floating-point weights as input and applies linear mapping to convert them into fixed-point numbers. These quantized numbers are then stored using binary two’s complement representation. The quantization process with *b* bits is given by **Equation 1**:


(1) $$w_{q}\in \mathbb{Z}|\;-2^{b-1}\leq w_{q}\leq 2^{b-1}-1$$

Here, *w* represents the value of each parameter in the tensor. Through linear mapping, VQK scales the floating-point weight values to binary integers with a specified number of bits, ensuring that the range of floating-point numbers aligns with the range of binary integers. This allows for efficient computation and memory storage using integers, ultimately improving computational efficiency.

#### 3.1.2 Distributed shifting

The distributed shifting adjusts the values in VQK through the scaling and biasing of KDS and CDS, ensuring that the output matches the original weight tensor. By setting the scaling factors and bias terms as ξ , $\xi_{s}$ , ( ϕ , and $\phi_{s}$ , and initializing the tensor using the L2 minimization criterion, the network can achieve optimal performance. For the initialization of the KDS tensor, the element-wise multiplication of the tensor approximates the original values, as shown in **Equations 2**–**4**:


(2) $$w_{0}\xi +\xi_{s}\approx w_{q0}$$

(3) $$w_{1}\xi +\xi_{s}\approx w_{q1}$$

(4) $$w_{BLK-1}\xi +\xi_{s}\approx w_{BLK-1}$$

We take the average value of ξ , denoted as $\hat{\xi }$ , and use the L2 criterion to minimize the initialization of the KDS tensor. Here, $w_{i}(i=1,2,\ldots ,BLK-1)$ represents the weight parameters of the quantized VQK tensor, and $w_{q_{i}}(i=1,2,\ldots ,BLK-1)$ represents the weight parameters of the original convolution tensor. The representation is given by **Equation 5**:


(5) $$\xi =\underset{\hat{\xi }}{\min}\sum_{i=0}^{BLK-1}{\left(w_{q_{i}}\hat{\xi }-w_{i}\right)}^{2}$$

DSConv dynamically adjusts the offset of the convolution kernel, enabling flexible adaptation to complex geometric shapes in images. The core of this approach lies in utilizing these offsets to finely control the convolution operation, thereby significantly enhancing the model’s ability to perceive and recognize targets of varying shapes and sizes. The representation is given by **Equation 6**:


(6) $$K_{i\pm c}=(x_{i}\pm c,y_{i}+\text{Δ}y)$$

In this context, $K_{i\pm c}$ represents the dynamic adjustment of the convolution kernel at position *i*, *x_i_* denotes the horizontal coordinate position of the current convolution kernel during the convolution operation, *y_i_* represents the vertical coordinate position, and *c* indicates the offset from the kernel’s center. Δ*y* is a learnable displacement that dynamically adjusts the shape of the convolution kernel to adapt to the complex geometric structure of the target. As shown in **Figure 2**, compared to the YOLOv8 network, DSConv pays more attention to the shape of small vascular bundles and exhibits better suppression of background noise, thus improving the comprehensiveness and accuracy of detection.

### 3.2 Multi-scale feature fusion method module

This paper proposes a detection strategy based on multi-scale feature fusion to address the challenges of capturing small targets, insufficient feature representation, and significant differences in multi-scale targets in rice stem cross-sectional small vascular bundles. The model’s ability to perceive small target details and understand complex semantic information is enhanced by effectively combining upsampling operations, convolutions, and the design of multi-scale detection heads. The improved network architecture is shown in **Figure 2**. The specific improvements are as follows:

#### 3.2.1 Introduction of upsampling operations to address model limitations in small object detection

In the Neck network’s Feature Pyramid Network(FPN) structure, we have designed and constructed three upsampling modules, F2, F3, and F4, corresponding to different feature scales in the FPN. These modules generate high-resolution feature maps through upsampling and concatenate them layer by layer with the shallow feature maps in the Backbone network, specifically P2-F2, P3-F3, and P4-F4. This concatenation strategy aims to fully integrate the spatial details in shallow features with the semantic information in deep features, enhancing the model’s ability to perceive and learn detailed features, thus significantly improving the model’s performance in small target detection tasks. The formula is given by **Equation 7**:


(7) $$F_{up}^{l}=U(F^{l})⊕F_{\text{s}\;}^{l}$$

where *F^l^* represents the *l*-th feature map from the deep features. U(.) denotes the upsampling operation, *F*_s_ represents the feature map from the shallow layers, and ⊕ denotes the concatenation operation.

#### 3.2.2 Introduction of downsampling convolution to enhance the model’s understanding of complex semantic information

In the Neck network’s PAN structure, we have constructed three convolution modules, T2, T4, and T5, corresponding to different scale feature levels in the PAN structure. Specifically, by fusing features from F2-T2, F4-T4, and P5-T5, we strengthen the top-down feature propagation path. This strategy effectively compensates for the traditional limitation of relying solely on FPN, where target localization information might be lost, thus improving the model’s semantic understanding and object detection performance in complex scenarios. The formula is given by **Equation 8**:


(8) $$F_{\text{down }}^{l}=C(F^{l})⊗F^{l-1}$$

where C(.) denotes the convolution operation, typically a downsampling convolution, *F^l^* ^−1^ represents the feature map from the previous layer, and ⊗ denotes the feature fusion operation.

#### 3.2.3 Addition of detection head to enhance the model’s detection capability for small objects

We introduced an additional detection head on top of the original network, which was designed to efficiently fuse features from different scales and enhance the model’s perception of multi-scale objects. By introducing detection heads specifically designed for different scales, the detection accuracy was significantly improved, especially in the tasks of small target detection and multi-scale segmentation, demonstrating outstanding performance. This improvement effectively compensates for the potential limitations of the original network in detecting small-scale targets and provides a more comprehensive and accurate solution for multi-scale object detection tasks. The formula for the detection output is given by **Equation 9**:


(9) $$O=D(F_{c})$$

where $F_{c}={⊕}_{i}F_{i}$ represents the concatenation of feature maps from different scales. D(.) denotes the detection head, which includes convolution, non-linear activation (such as SiLU), and loss computation. *O* is the output, which includes the object classification scores and bounding box regression.

Our approach enhances the model’s detection capability for multi-scale objects by employing a multiscale feature fusion strategy, which integrates upsampling operations, downsampling convolutions, and the design of multi-scale detection heads. This comprehensive approach provides an effective solution for accurate object detection in complex scenarios.

### 3.3 PIoU loss function

Boundary box regression (BBR) loss function is also crucial in the detection of small vascular bundles in rice stem’s cross-sections. A well-designed boundary loss function might bring significant performance improvements to the model. YOLOv8 calculates the boundary box regression loss using Complete Intersection over Union (CIoU). CIoU takes into account three important aspects when calculating the boundary box regression loss, i.e., the overlap area, the distance between the centers, and the aspect ratio. Given a predicted bounding box *b* and a ground truth bounding box *b^gt^*, the CIoU loss function is given by **Equation 10**:


(10) $$L_{\text{CIoU}}=1-IoU+\frac{\rho^{2}(b,b^{gt})}{c^{2}}+\alpha v$$

In this context, IoU refers to Intersection over Union, $\rho^{2}(b,b^{gt})$ is the squared Euclidean distance between the centers of the predicted bounding box *b* and the ground truth bounding box *b^gt^*. *c* represents the diameter of the smallest enclosing box that contains both the predicted and ground truth bounding boxes. *α* is a weight coefficient, and *v* is the aspect ratio consistency penalty term.

However, the CIoU loss function fails to fully account for differences in target scales during the calculation process. This issue becomes particularly apparent when handling small and large targets, where the localization accuracy for small targets is often insufficient. This limitation could lead to a decline in small target detection performance, thus impacting the overall detection accuracy. To address this issue, this paper introduces the PIoU Khalili and Smyth (2024) loss function to replace the CIoU loss function in the original network. PIoU, while measuring the overlap area between the predicted and ground truth boxes, further incorporates a penalty mechanism by minimizing the Euclidean distance between the corner points of the predicted and ground truth boxes. This improves the model’s ability to capture the positional relationship between the targets. Additionally, this loss function is more effective in balancing detection performance across targets of different scales, particularly demonstrating superior performance in small target detection. The formula is given by **Equation 11**:


(11) $$L_{\text{PIoU}}=3\cdot (\lambda q)\cdot e^{-{\left(\lambda q\right)}^{2}}\cdot (1-IoU-e^{-P^{2}})$$

The penalty term *P* is given by **Equations 12**–**16**:


(12) $$dw_{1}=|(b1_{x2}-b1_{x1})-(b2_{x2}-b2_{x1})|$$

(13) $$dw_{2}=|(b1_{x2}-b1_{x1})+(b2_{x2}-b2_{x1})|$$

(14) $$dh_{1}=|(b1_{y2}-b1_{y1})-(b2_{y2}-b2_{y1})|$$

(15) $$dh_{2}=|(b1_{y2}-b1_{y1})+(b2_{y2}-b2_{y1})|$$

(16) $$P=\frac{1}{4}(\frac{dw_{1}+dw_{2}}{w_{gt}}+\frac{dh_{1}+dh_{2}}{h_{gt}})$$

where *b*_1_ and *b*_2_ represent the coordinates of the predicted and ground truth boxes, with (*x*_1_ *,y*_1_) denoting the top-left corner coordinates, and (*x*_2_ *,y*_2_) representing the bottom-right corner coordinates. *w_gt_* is the width of the ground truth box, and *h_gt_* is the height of the ground truth box. IoU is the Intersection over Union between the predicted and ground truth boxes. *d* represents the Euclidean distance between the corresponding corner points of the predicted and ground truth boxes. *λ* is the weight coefficient for the penalty term, which adjusts the influence of *P* on the loss. *q* is the focusing factor, scaled by the exponent of *P*, and is expressed as *q* = *e* ^−^ *^P^* ^2^.

The PIoU loss enhances the model’s ability to model the spatial relationship between object locations by introducing a penalty mechanism based on the Euclidean distance of corner points. It effectively balances the detection performance across different scales of targets, particularly significantly improving the localization accuracy and robustness in small object detection.

## 4 Experiments

To evaluate the performance of the proposed method in the microscopic image analysis of small vascular bundles in rice stem’s cross-sections, this study conducted a comprehensive assessment of the RiceSVBDete method. Using a custom dataset, we performed a series of extensive experiments aiming at evaluating the effectiveness of Rice-SVBDete in accurately identifying small vascular bundles.

### 4.1 Experiment setup

#### 4.1.1 Datasets

**Figure 3:**
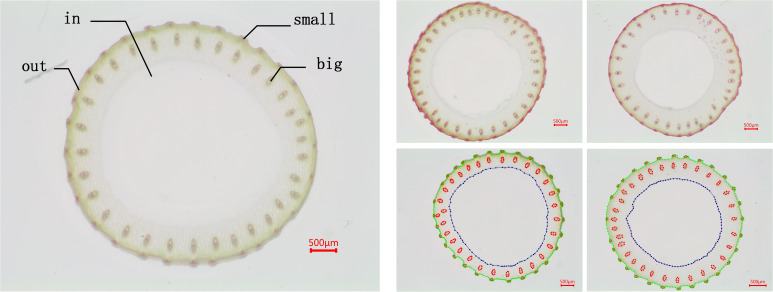
Rice stem cross-sections microscopic image.

The dataset used in our experiments was provided by Guangxi University, comprising core germplasm resources selected from the 3K RGP (3,000 Rice Genomes Project) Wang et al. (2018). This core collection exhibits extensive genetic diversity and serves as a representative subset of global rice germplasm resources. The materials originate from a wide range of geographic regions, ensuring high representativeness. They have been widely adopted by multiple research institutions, including Guangxi University, for studies on rice genetic improvement and gene discovery, highlighting their substantial scientific and practical value. From this core collection, A total of 289 germplasm accessions from different countries with similar heading dates were selected based on their genetic diversity. These included 146 indica rice accessions and 99 japonica rice accessions (38 subtropical japonica, 13 tropical japonica, 36 temperate japonica from Southeast Asia, and 12 GJ-adm). Additionally, 23 japonica rice accessions (cA) and six japonica rice accessions (cB) from South Asia, along with 15 admixture varieties (admix), were included to represent the major temperate and subtropical rice gene pools. The rice stem’s cross-sections were collected from the second internode at the base of the stem during the heading stage. The sections were sliced to approximately 0.2–0.5 mm thickness and stored as TIF images. The resulting dataset comprises 1091 microscopic images containing 66728 labeled instances of four distinct feature types. **Table 1** shows the distribution of images and labeled cases for each feature type. Using the Labelme (version 5.2.1) tool, the contours of small vascular bundles (small), large vascular bundles (big), cavities (in), and stem perimeters (out) were annotated for each image. The annotation results are shown in **Figure 3**, **4**. The dataset was divided into training, validation, and test sets at a ratio of 8:1:1, which were used for model training, validation, and testing, respectively.

**Table 1:** Statistics of rice stem’s cross-sections microscopic image annotation dataset.

Dataset	Small	Big	In	Out
Boxes	30854	33692	1091	1091
Images Total	1091			
Boxes Total	66728			

**Figure 4:**
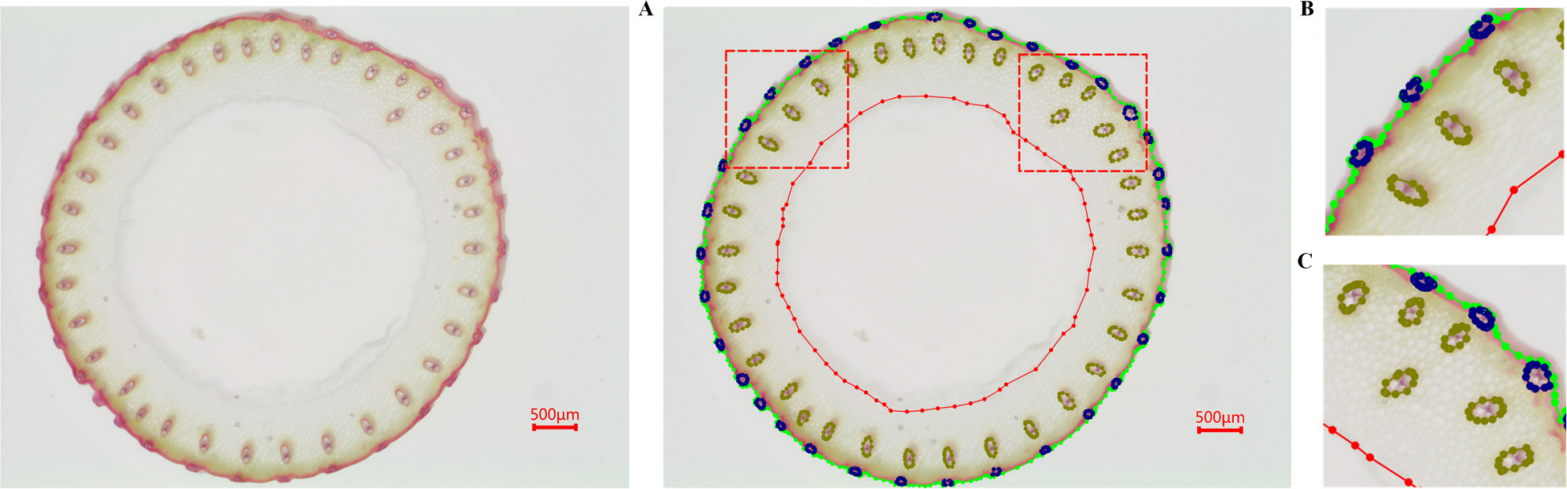
Dataset annotation. **(A)** shows the overall annotation result, while **(B, C)** are zoomed-in views of specific regions in **(A)**.

#### 4.1.2 Implementation detail

We implemented the Rice-SVBDete method based on the YOLOv8 framework of the PyTorch deep learning platform, training the model on an NVIDIA GeForce RTX 3090 GPU with 24GB of memory. The model was trained for 100 epochs using the Adam optimizer, with a learning rate of 0.01, a batch size of 16, and an input image size of 640×640. In our implementation, we adopted a four-scale anchoring system: P2/4, P3/8, P4/16, and P5/32. Specifically, the P2/4 scale anchors were designed for detecting small objects, P3/8 and P4/16 anchors targeted medium-sized objects, and P5/32 anchors were tailored for detecting large objects. This hierarchical structure ensures comprehensive coverage of object sizes within microscopic images.

#### 4.1.3 Evaluation metrics

To evaluate the Rice-SVBDete algorithm’s performance comprehensively, we select four evaluation metrics: precision(P), Recall(R), Mean Average Precision (MAP), Mean Absolute Percentage Error (MAPE), Root Mean Square Error (RMSE), Dice coefficient(Dice), and Intersection over Union (IoU). These metrics evaluate the algorithm’s ability to accurately identify and classify the feature cells present in microscopic images.

Precision denotes the ratio of true positive cases predicted to be true to all predicted positive cases Liu et al. (2020). It is calculated as shown in **Equation 17**:


(17) P=TP/(TP+FP)

where TP denotes that the predicted value is the same as the true value, and the predicted value is a positive sample; FP denotes that the predicted value is different from the true value, and the predicted value is a positive sample.

Recall denotes the ratio of true positive cases predicted to be true to all true positive cases. It is calculated as shown in **Equation 18**:


(18) R=TP/(TP+FN)

where FN denotes that the predicted value is not the same as the true value and the predicted value is a negative sample.

The AP curve is the area surrounded by the curve in two dimensions: Precision and Recall. Usually, Precision is higher when Recall is lower and lower when Recall is higher. That is, the larger the AP curve, the better the model’s performance. The definition of AP is given by **Equation 19**:


(19) $$AP=\int_{0}^{1}\;\text{P}(\text{R})d(R)$$

MAP is a comprehensive evaluation metric focusing on sequence weights. It has become one of the most important practical metrics for image recognition problems in recent years. mAP@.5 indicates that the average AP of all images under each category is calculated at IoU=0.5, and the higher the value of mAP, the better the model’s performance. The definition of mAP is given by **Equation 20**:


(20) $$mAP=\frac{1}{N}\sum_{i=1}^{N}{\text{AP}}_{i}$$

where *AP_i_* represents the average precision value for the category indexed by *i*, and *N* denotes the total number of categories in the training dataset. mAP@.5 is the average precision calculated at an IoU threshold of 0.5. mAP@.5:.95 is calculated across IoU thresholds from 0.5 to 0.95, with values computed at intervals of 0.05.

Mean Absolute Percentage Error (MAPE) Nendel et al. (2023) is a metric used to measure the error between predicted and actual values, particularly in regression tasks. It represents the average percentage error, with lower values indicating smaller prediction errors. The definition of MAPE is given by **Equation 21**:


(21) $$MAPE=\frac{1}{n}\sum_{i=1}^{n}|\frac{y_{i}-{\hat{y}}_{i}}{y_{i}}|\times 100$$

here, *y_i_* represents the *i*-th actual value, ${\hat{y}}_{i}$ denotes the *i*-th predicted value, and *n* is the total number of data points.

Root Mean Square Error (RMSE) Nendel et al. (2023) is a commonly used metric for evaluating the error between predicted and actual values. It emphasizes larger errors and is more sensitive to outliers. A smaller RMSE value indicates higher prediction accuracy. The definition of RMSE is given by **Equation 22**:


(22) $$RMSE=\sqrt{\frac{1}{n}\sum_{i=1}^{n}{\left(y_{i}-{\hat{y}}_{i}\right)}^{2}}$$

The Dice coefficient (Dice), a commonly used similarity metric, is widely applied in image segmentation tasks to evaluate the degree of overlap between predicted and ground truth segmentations.

Intersection over Union (IoU), another frequently used performance metric for image segmentation, quantifies the ratio of the intersection to the union of the predicted and ground truth segmentation regions.

### 4.2 Comparisons with state-of-the-art methods

To identify the most suitable baseline model, a comparative evaluation was conducted under a consistent evaluation protocol. Representative two-stage method Faster R-CNN Ren et al. (2016) and one-stage methods SSD Liu et al. (2016), RetinaNet Lin et al. (2017), and YOLOv8 Jocher et al. (2023) were selected for comparison. The results are presented in **Table 2**.

**Table 2:** Comparison of basic models.

Method	SSD			RetinaNet			Faster R-CNN			YOLOv8		
	*P* (%)	*R*(%)	*F*1	*P* (%)	*R*(%)	*F*1	*P* (%)	*R*(%)	*F*1	*P* (%)	*R*(%)	*F*1
small	97.18	36.39	0.53	97.08	32.49	0.49	91.49	43.23	0.59	61	57	0.59
in	60	6.74	0.12	0	0	0	50	25.84	0.34	99.1	1	0.99
big	98.9	32.53	0.49	97.95	31.09	0.47	97.41	36.64	0.53	83.6	84.9	0.84
out	0	0	0	0	0	0	0	0	0	99.1	1	0.99

As shown in **Table 2**, while methods such as SSD, RetinaNet, and Faster R-CNN exhibit high *P* on small vascular bundle detection, their *R* and F1 scores remain relatively low. For instance, RetinaNet achieves a precision of 97.08% but only 32.49% in *R*, yielding an F1 score of 0.49. In contrast, YOLOv8 achieves a balanced performance with a *P* of 61%, a *R* of 57%, and an F1 score of 0.59 on small targets. Furthermore, for inner-region detection—where precise localization is critical—YOLOv8 significantly outperforms all other methods, achieving nearly perfect detection performance (F1 score = 0.99). These findings suggest that YOLOv8 strikes an optimal balance between detection accuracy and efficiency, particularly under the challenging conditions of dense distribution, blurred boundaries, and low contrast, which are typical in rice stem cross-sectional imaging. Therefore, we selected YOLOv8 as the base model for our Rice-SVBDete framework due to its superior capability in capturing small and intricate anatomical features while maintaining computational efficiency.

To evaluate the effectiveness of our proposed Rice-SVBDete method, we compared it against several widely used state-of-the-art image recognition algorithms. Specifically, we benchmarked our method against YOLOv8 Jocher et al. (2023), ASF-YOLO Kang et al. (2024), SOD-YOLO Khalili and Smyth (2024), and Subtle-YOLO Zhao et al. (2024). These algorithms represent diverse architectural paradigms and have demonstrated exceptional performance in various computer vision tasks, providing a robust baseline for comparative analysis.

**Figure 5:**
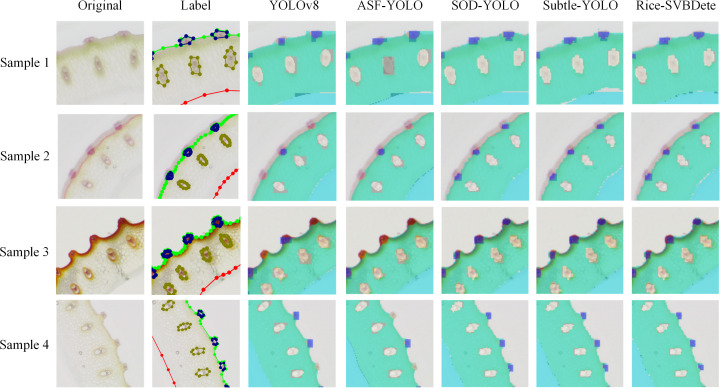
Detection results of State-of-the-Art Methods.

**Table 3** presents the quantitative results of the comparative analysis. As shown in the table, our proposed Rice-SVBDete method outperforms all state-of-the-art methods across all four evaluation metrics. Specifically, Rice-SVBDete achieves an impressive *P* of 0.794 and *R* of 0.784, surpassing SOD-YOLO by 0.013 and 0.015, respectively. Additionally, Rice-SVBDete attains the highest mAP@.5 of 0.732, outperforming its closest competitor, SOD-YOLO, by 0.017. Rice-SVBDete also demonstrates superiority in the most challenging metric, mAP@.5:.95, achieving a score of 0.248, which is 0.011 higher than the second-best method, the improved YOLOv8. These results underscore the effectiveness of our proposed method in accurately detecting and localizing objects under varying degrees of occlusion and overlap. Moreover, we illustrate the detection results of Rice-SVBDete in **Figure 5**. The figure clearly demonstrates that Rice-SVBDete successfully identifies small vascular bundles of varying sizes and accurately detects structures with blurred boundaries.

**Table 3:** Comparisons with state-of-the-art methods.

Method	Precision	Recall	mAP@.5	mAP@.5:.95	Dice	IoU
YOLOv8 Jocher et al. (2023)	0.61	0.57	0.501	0.187	0.589	0.418
SOD-YOLO Khalili and Smyth (2024)	0.781	0.769	0.715	0.237	0.775	0.633
ASF-YOLO Kang et al. (2024)	0.632	0.588	0.531	0.194	0.609	0.438
Subtle-YOLO Zhao et al. (2024)	0.765	0.747	0.703	0.235	0.756	0.608
Rice-SVBDete	0.794	0.784	0.732	0.248	0.789	0.651

**Figure 6:**
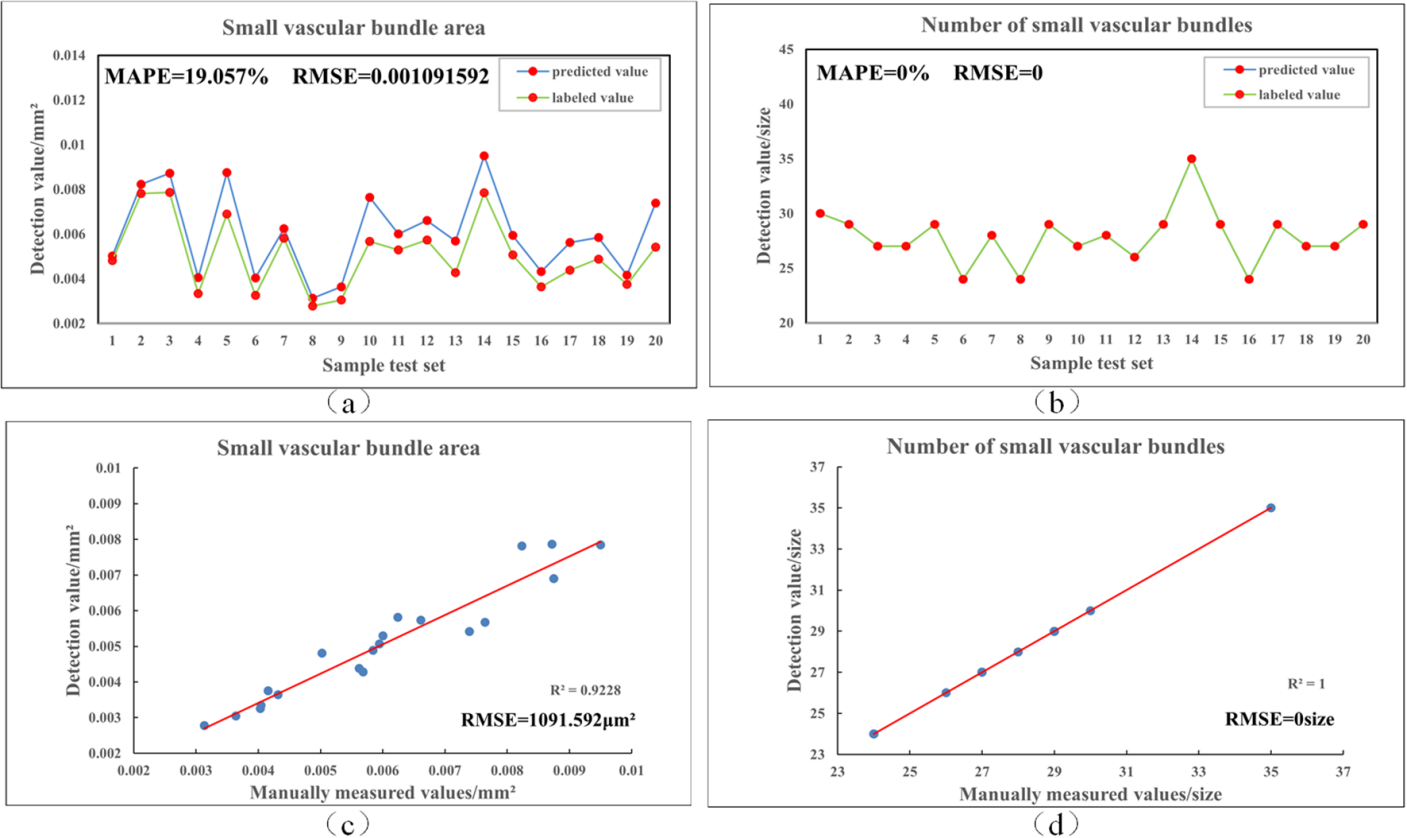
Comparison of manual labeling and algorithmic detection results. **(a, b)** show the fitting of area and count of small vascular bundles between the predictions and the labels, **(c, d)** present the least squares fitting results for these two parameters.

To further validate the prediction accuracy of Rice-SVBDete, we used manually annotated vascular bundle regions as the reference standard and performed a comparative analysis between the algorithm’s predicted results and the manual measurements. The analysis results are shown in **Figure 6**. As observed in the figure, the algorithm demonstrates high accuracy in predicting the number and area of small vascular bundles, with MAPE values of 0.0% and 19.06%, respectively, and RMSE values of 0 and 0.001. These results indicate that Rice-SVBDete exhibits high reliability and accuracy in predicting small vascular bundle parameters.

### 4.3 Ablation studies

#### 4.3.1 Effectiveness of different modules

We also conducted a comprehensive ablation study to evaluate each proposed module’s impact. Specifically, we systematically included or excluded the DSConv, MFF, and PIoU modules from the model and assessed their performance. The experimental results in **Table 4** clearly show improved model performance with the addition of more modules. Incorporating all three modules (DSConv, MFF, and PIoU) resulted in the highest *P*, *R*, mAP@.5, and mAP@.5:.95. This suggests a synergistic effect between DSConv, multi-scale feature fusion, and PIoU loss. The consistent improvement across all evaluation metrics highlights the crucial role of multi-scale feature fusion in enhancing object detection accuracy.

**Table 4:** Experimental results using DSConv only, MFF only, and DSConv+MFF.

DSConv	MFF	PIoU	Precision	Recall	mAP@.5	mAP@.5:.95
			0.61	0.57	0.501	0.187
✓			0.617	0.574	0.513	0.187
	✓		0.784	0.765	0.717	0.248
		✓	0.618	0.579	0.512	0.189
✓	✓		0.773	0.761	0.705	0.235
	✓	✓	0.767	0.765	0.709	0.242
✓		✓	0.603	0.567	0.501	0.187
✓	✓	✓	0.794	0.784	0.732	0.248

The symbol ✓ means that the module is selected.

#### 4.3.2 Effectiveness of DSConv

To evaluate the effectiveness of the DSConv module, we trained our model and conducted extensive experiments. **Table 4** highlights the significant impact of DSConv on the model’s performance metrics. The *P* and *R* values increased from 0.61 and 0.57 without DSConv to 0.617 and 0.574 with DSConv, respectively, indicating that this module enhances feature extraction capability while minimizing false positives and false negatives. Moreover, the mAP@.5, evaluated at an IoU threshold of 0.5, showed a significant improvement, increasing from 0.501 without DSConv to 0.513 with DSConv.

#### 4.3.3 Effectiveness of MFF

**Figure 7:**
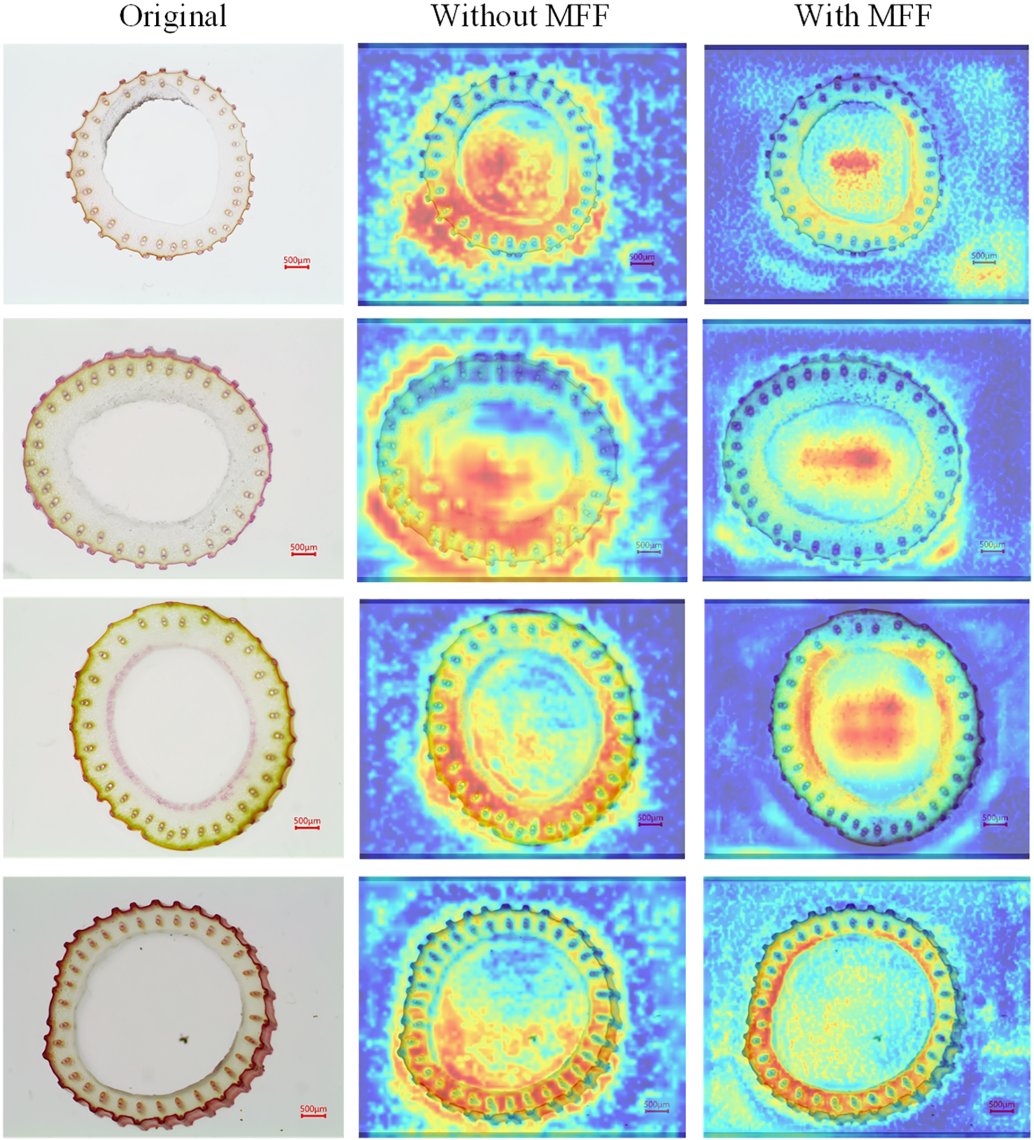
Heatmap examples without MFF and with MFF.

The MFF module represents a significant advancement in addressing the complex challenges associated with the small vascular bundles in rice stem’s cross-sections, such as their highly variable morphology, dense arrangement, indistinct edges, and low contrast. This module integrates the strengths of both shallow and deep feature representations within the model. The heatmap in **Figure 7** provides a visual representation of the impact of the MFF module. After incorporating the MFF module, a focused and precise attention map was obtained, highlighting the model’s capability to detect small vascular bundles with varying morphological features, including those with blurred boundaries. Overall, the MFF module addresses the limitations of single-scale models, which struggle to balance global semantic information and local detailed representation. By leveraging the complementarity of features across different scales, the MFF module enhances the model’s ability to perceive complex scenes on a global scale while improving its capacity to capture small targets and fine details. Consequently, it significantly enhances Rice-SVBDete’s performance in accurately detecting and analyzing small vascular bundles.

**Figure 8:**
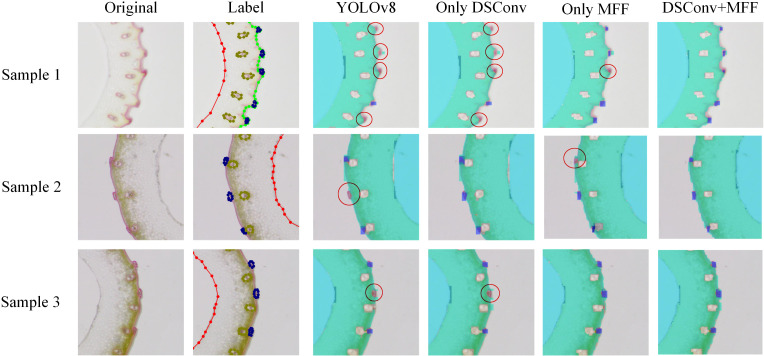
Visualization of detection results before and after applying DSConv and MFF modules.

**Figure 8** presents a qualitative comparison of vascular bundle detection results across different model configurations, including the baseline YOLOv8, and models with individual or combined enhancements: DSConv, MFF, and DSConv+MFF. Three representative samples are shown, each with the original image, ground truth annotations, and detection results. Red circles highlight incorrect or missed detections. In the YOLOv8 baseline, several small or low-contrast vascular bundles are either missed or inaccurately localized (e.g., Samples 1, 2, and 3). Introducing DSConv improves detection in regions with subtle edge information by enriching local spatial features. MFF further enhances multi-scale context awareness, helping to recover small or clustered targets. However, each module alone still suffers from occasional false negatives or localization inaccuracies. The combined model (DSConv+MFF) significantly reduces both missed detections and localization errors. As shown in the final column, most vascular bundles are correctly identified across all samples, even in challenging regions where the baseline model fails. These results demonstrate that the proposed structural enhancements improve quantitative performance and provide visibly more accurate and robust detections, especially for small, ambiguous targets prone to failure in the original YOLOv8 model.

#### 4.3.4 Model efficiency analysis

To evaluate the computational efficiency of the proposed method, we conducted a detailed runtime analysis across various ablated versions of the model.

As shown in **Table 5**, the baseline YOLOv8 model achieves the lowest per-image processing time of 12.8 ms, attributed to its lightweight architecture. However, the integration of individual modules—DSConv, MFF, and PIoU—inevitably leads to increased inference times. When DSConv is added alone, the inference time rises to 29.0 ms, mainly due to the dynamic kernel operations introduced by depthwise separable convolutions. Adding MFF results in a moderate increase to 19.2 ms, reflecting the cost of enhanced multi-scale feature fusion. Similarly, incorporating PIoU increases the runtime to 16.8 ms by introducing a more refined localization strategy. Combined configurations present a more complex picture. Including both DSConv and MFF yields the highest inference time among all variants (31.5 ms), while DSConv + PIoU and MFF + PIoU combinations result in 24.3 ms and 15.5 ms per inference, respectively. These combinations reflect trade-offs between feature richness and computational cost. Notably, the proposed full model—comprising all three modules: DSConv, MFF, and PIoU—achieves a total per-image runtime of 37.7 ms. While this represents the highest latency among all configurations, it still supports an effective throughput of approximately 26 FPS. This level of performance remains sufficient for most offline or near real-time agricultural scenarios, and is justified by the substantial improvements in detection accuracy delivered by the synergistic effect of the three modules.

**Table 5:** Time consumption for DSConv only, MFF only, and DSConv+MFF.

DSConv	MFF	PIoU	Preprocess	Inference	Postprocess	Per image
			0.8	9.5	2.5	12.8
✓			0.6	29	2.8	32.4
	✓		0.9	17	1.3	19.2
		✓	0.9	14	1.9	16.8
✓	✓		0.5	31.5	2.9	34.9
	✓	✓	0.8	15.5	9.0	25.3
✓		✓	1.0	24.3	2.5	27.8
✓	✓	✓	0.5	36.4	0.8	37.7

The symbol ✓ means that the module is selected, and the time unit is ms.

### 4.4 Effectiveness of other parameters

To further evaluate the adaptability and effectiveness of the Rice-SVBDete method, we conducted detections on parameters such as the large vascular bundles (big), cavities (in), and stem perimeter (out) of rice stem’s cross-sections. The core metrics, including *P*, *R*, and mAP, were recorded, as shown in **Table 6**. The experimental results demonstrate significant improvements in detecting large vascular bundles, cavities, and stem perimeter using the Rice-SVBDete method, specifically for the “in” category, *P*, and mAP@.5:.95 improved by 0.004 and 0.001, respectively. In the “big” category, notable enhancements were observed in *P*, *R*, mAP@.5, and mAP@.5:.95, with increases of 0.147, 0.135, 0.173, and 0.15, respectively. For the “out” category, *P* and mAP@.5:.95 improved by 0.004 and 0.002, respectively. These results fully demonstrate that the Rice-SVBDete method exhibits excellent performance in detecting small vascular bundles and achieves outstanding accuracy and robustness in detecting parameters such as large vascular bundles and cavities. This effectively enhances the method’s overall detection capability and adaptability.

**Table 6:** Detection results of other parameters.

Method	YOLOv8				Rice-SVBDete			
Metrics	P	R	mAP@.5	mAP@.5:.95	P	R	mAP@.5	mAP@.5:.95
in	0.991	1	0.995	0.991	0.995	1	0.995	0.992
big	0.836	0.849	0.809	0.326	0.983	0.984	0.982	0.476
out	0.991	1	0.995	0.906	0.995	1	0.995	0.908

## 5 Conclusion

The small vascular bundles in rice stem’s cross-sections exhibit characteristics such as highly variable shapes, dense arrangements, blurred edges, and low contrast, making them difficult to capture. These features present significant challenges for traditional detection and recognition methods. However, rice breeding, quality assessment, and related biological research require advanced and reliable automated identification technologies. Deep learning-based methods, especially the development of artificial neural networks, provide a highly promising solution for the automated detection of small vascular bundles in rice stem’s cross-sections. In this study, we propose a new method, Rice-SVBDete, by introducing DSConv to optimize the feature extraction process and integrating an MFF module to enhance the model’s ability to express diverse features and overall accuracy, effectively addressing the key issue of automatic detection of small vascular bundles in rice. The Rice-SVBDete method precisely captures the edge details of small vascular bundles while effectively overcoming detection bias caused by low contrast and noise interference.

Although the method builds upon recent deep learning components, its novelty lies in the context-aware integration and adaptation of these techniques for biological imaging tasks. Specifically, the architectural design is tailored to the unique challenges of detecting small and ambiguous structures in complex plant tissue environments, which has been rarely addressed in existing literature. In experiments, Rice-SVBDete outperforms existing state-of-the-art methods in core metrics such as *P*, *R*, and mAP, fully validating its excellent performance and broad adaptability in small vascular bundle detection tasks, providing a reliable and efficient solution for rice stem cross-section analysis.

However, the method has certain limitations. It relies heavily on high-quality annotated datasets for training, which may not be readily available for different crop species with distinct structural characteristics. Future work will focus on extending the model’s adaptability to a broader range of crop species by incorporating cross-species transfer learning and domain generalization strategies. Efforts will also aim to reduce the dependency on annotated data through self-supervised or weakly-supervised learning techniques. Additionally, integrating multimodal imaging data, such as hyperspectral or X-ray imaging, will be explored to improve robustness and accuracy, enabling more comprehensive microscopic image parameters detection across diverse agricultural applications.

## Data Availability

The raw data supporting the conclusions of this article will be made available by the authors, without undue reservation.
